# Accuracy and Reliability of Dental Age Estimation Methods in Iranian Children: A Systematic Review and Meta‐Analysis

**DOI:** 10.1155/bmri/9351167

**Published:** 2026-06-14

**Authors:** Razieh Jabbarian, Mehdi Ranjbaran, Aida Mokhlesi

**Affiliations:** ^1^ Department of Pediatric Dentistry, Qazvin University of Medical Sciences, Qazvin, Iran, qums.ac.ir; ^2^ Department of Epidemiology and Biostatistics, Non-Communicable Diseases Research Center, Research Institute for Prevention of Non-Communicable Diseases, Qazvin University of Medical Sciences, Qazvin, Iran, qums.ac.ir; ^3^ Social Determinants of Health Research Center, Research Institute for Prevention of Non-Communicable Diseases, Qazvin University of Medical Sciences, Qazvin, Iran, qums.ac.ir; ^4^ USERN Office, Qazvin University of Medical Sciences, Qazvin, Iran, qums.ac.ir; ^5^ Student Research Committee, School of Dentistry, Qazvin University of Medical Sciences, Qazvin, Iran, qums.ac.ir; ^6^ Network of Interdisciplinarity in Neonates and Infants (NINI), Universal Scientific Education and Research Network (USERN), Tehran, Iran, usern.tums.ac.ir

**Keywords:** chronological age, Demirjian′s method, dental age estimation, Iranian children, meta-analysis, systematic review

## Abstract

**Objectives:**

This study is aimed at systematically evaluating the accuracy and reliability of dental age (DA) estimation methods and conducting a meta‐analysis of studies on different DA estimation methods in Iranian children.

**Methods:**

A systematic search of English and Persian literature from database inception to March 2024 was conducted across international and national databases to identify eligible studies on DA estimation using radiographs in Iranian children. Studies that used dental radiographs from healthy individuals aged 3–18 years and reported the mean difference (MD) between DA and chronological age (CA) were included. The meta‐analysis calculated the MD between dental and chronological age, with 95% confidence intervals (CIs), to assess the applicability of the DA method for estimating CA in the Iranian population.

**Results:**

Twenty‐seven eligible published articles were included in this study. Demirjian′s method overestimated age (overall MD: males: +0.784 years; females: +0.323 years). Among the alternative methods studied in the systematic review, Moorrees and Nolla′s methods tended to underestimate age, whereas Cameriere, Smith, and London Atlas′methods tended to overestimate age.

**Conclusion:**

Most methods studied consistently overestimated age and should be used cautiously in forensic age estimation, underscoring the importance of tailoring methods such as the Demarjian method for the Iranian population to ensure accuracy and relevance in professional practice. Additional studies employing similar methodologies and focusing on the customization of dental age estimation methods are recommended to improve accuracy and applicability across diverse populations.

## 1. Introduction

Age determination is essential for accurate diagnosis and treatment planning in orthodontics and pediatric dentistry [1–3]. Several indicators of physical growth and development, such as skeletal and dental age (DA), menopause, physical and sexual maturity, height, and weight, can be used to estimate chronological age (CA) [4–6]. Among these, DA is exceptionally reliable due to its minimal susceptibility to local factors and its applicability across a broad age range via radiographic assessment [7–9].

In recent decades, several methods have been developed to assess DA in children, primarily by radiography [10]. The methods used to estimate the DA of children are based on the methods of Nolla [11], Moores [12], Demirjian [11–28], Willem [2, 28–31], Smith [2, 28], London Atlas [2], and Cameriere [1, 2, 28–30]. In the first six methods, the developmental stage of teeth is determined based on their degree of mineralization. However, in Cameriere′s method, the relationship between age and the size of the apical foramen in the tooth is evaluated in seven lower left permanent teeth [10]. Several studies have examined the accuracy of different methods for estimating DA and its agreement with CA [1, 2, 13, 32]. Some dental parameters related to diet change and economic, social, and genetic factors have also been proposed. The standards for tooth eruption vary across populations over time [33–35]. Despite numerous studies evaluating DA estimation methods in Iran, there remains a lack of synthesized evidence regarding their comparative accuracy and reliability in Iranian children. To the authors′ knowledge, no systematic review and meta‐analysis have comprehensively assessed the performance of these methods in this population.

Despite several separate studies aiming at the most accurate DA estimation methods, to the authors′ knowledge, there is no systematic review and meta‐analysis on DA estimation in Iran. Therefore, this study is aimed at systematically reviewing and performing a meta‐analysis of the available studies on DA estimation methods applicable to Iranian children.

The review seeks to evaluate the accuracy and reliability of these methods, identify the most suitable approaches for the Iranian population, and provide evidence‐based recommendations for clinical and forensic applications.

## 2. Materials and Methods

### 2.1. Study Design and Ethical Aspects

A systematic literature review and meta‐analysis were performed according to the Preferred Reporting Items for Systematic Reviews and Meta‐Analyses (PRISMA) guidelines and the Cochrane standards. The protocol for this systematic review and meta‐analysis was registered in the International Prospective Register of Systematic Reviews in June 2022 (Identification Number: CRD42022336041).

### 2.2. Research Question

The systematic research question was developed based on PECO:

• P (population): Iranian children (3–18 years).
• E (exposure): DA estimation methods.
• C (comparison): CA.
• O (outcome): Accuracy of DA estimation methods.
– Calculating the mean difference (MD) between CA and DA in each method in general and based on gender.
– “Which of the DA estimation methods (I) is more accurate to estimate the CA (C) of the Iranian children (P)?”

### 2.3. Sources of Information and Search Strategy

We searched international and national databases, including PubMed, Scopus, Web of Science, Google Scholar, SID, Magiran, and IDM, using combinations of English and Persian keywords (Table 1). The national databases were searched using all possible combinations of Persian equivalents of identified keywords. The reference lists of all initially retrieved studies and review articles were manually searched to identify additional studies that might have been missed. The database search covered the period from database inception to March 2024. The initial search was conducted in June 2022 and updated in March 2024. Studies published before 2006 were rarely available in full text or lacked sufficient extractable data; therefore, 2006 was chosen as the lower limit to maximize study quality and comparability.

**Table 1:** Database search strategies.

Data base	Search details
Pubmed	(((“Forensic Dentistry”[MeSH Terms] OR “forensic odontology”[All Fields] OR “Forensic Sciences”[MeSH Terms] OR “Dentistry”[MeSH Terms] OR “Oral Medicine”[MeSH Terms] OR “odontology”[All Fields])) AND ((“Age Determination by Teeth”[MeSH Terms] OR “dental age”[Title/Abstract] OR “dental age estimation”[Title/Abstract] OR “dental formation”[Title/Abstract] OR “dental maturity”[Title/Abstract] OR “age estimation”[Title/Abstract] OR “age determination”[Title/Abstract]))) AND ((“iran”[All Fields] OR “iranian”[All Fields] OR “iranians”[All Fields] OR “iranian people”[All Fields] OR “iranian population”[All Fields]))
	(“iran”[All Fields] OR “iranian”[All Fields] OR “iranians”[All Fields] OR “iranian people”[All Fields] OR “iranian population”[All Fields])
	(“Age Determination by Teeth”[MeSH Terms] OR “dental age”[Title/Abstract] OR “dental age estimation”[Title/Abstract] OR “dental formation”[Title/Abstract] OR “dental maturity”[Title/Abstract] OR “age estimation”[Title/Abstract] OR “age determination”[Title/Abstract])
	(“Forensic Dentistry”[MeSH Terms] OR “forensic odontology”[All Fields] OR “Forensic Sciences”[MeSH Terms] OR “Dentistry”[MeSH Terms] OR “Oral Medicine”[MeSH Terms] OR “odontology”[All Fields])

Web of Science	#1 AND #2 and IRAN (Countries/Regions)
	ALL=(“Age Determination by Teeth” OR “dental age” OR “dental age estimation” OR “dental formation” OR “dental maturity” OR “age estimation” OR “age determination”)
	ALL=(“Forensic Dentistry” OR”forensic odontology” OR “Forensic Sciences” OR Dentistry OR “Oral Medicine” OR odontology)

Scopus	(ALL ((“Age Determination by Teeth” OR “dental age” OR “dental age estimation” OR “dental formation” OR “dental maturity” OR “age estimation” OR “age determination”"))) AND (ALL ((“forensic dentistry” OR “forensic odontology” OR “forensic sciences” OR “dentistry” OR “oral medicine” OR “odontology”"))) AND (AFFILCOUNTRY ((“iran”")))
	ALL ((“Age Determination by Teeth” OR “dental age” OR “dental age estimation” OR “dental formation” OR “dental maturity” OR “age estimation” OR “age determination"))
	ALL ((“forensic dentistry” OR “forensic odontology” OR “forensic sciences” OR “dentistry” OR “oral medicine” OR “odontology”"))

Sid	(Dental age) OR (Dental age eastimation) (in persion))
Magiran	(Dental age) OR (Dental age eastimation)(in persion))
Idm research	(Dental age) OR (Dental age eastimation) (in persion))

### 2.4. Eligibility Criteria

#### 2.4.1. Inclusion Criteria

The inclusion criteria were as follows: cross‐sectional or retrospective studies on healthy individuals without developmental anomalies who had all their permanent mandibular teeth (erupted or unerupted), studies reporting the mean and standard deviation of CA and DA in the Iranian children, articles published in English or Persian, articles published from their inception to March 2024, and availability of the full text.

#### 2.4.2. Exclusion Criteria

The exclusion criteria were as follows: duplicate publications; letters to the editor, case reports, review articles, and editorials; studies on patients with underlying medical conditions or developmental anomalies; and studies that did not report the mean and standard deviation for both chronological and DA, which were necessary for the meta‐analysis.

### 2.5. Selection of Studies

The studies were selected in three steps. In the first step, two authors separately performed the search. EndNote (Version 20.2.1) was used to import the articles and remove duplicates. A calibration exercise was then conducted to train the reviewers to select studies based on the eligibility criteria. In the second step, two reviewers independently evaluated the titles and abstracts of the articles based on the inclusion and exclusion criteria. Disagreements between the reviewers were discussed with a third reviewer to reach a consensus. In the third step, the full text of primarily eligible articles was read and evaluated for inclusion.

### 2.6. Risk of Bias of Eligible Studies

The risk of bias for each eligible study was assessed using the Joanna Briggs Institute (JBI) Critical Appraisal Tools for use in JBI Systematic Reviews for cross‐sectional studies. Each study was classified as high (< 49%), medium (50%–69%), or low (> 70%) risk of bias based on the percentage of positive answers to the questions of the JBI tool [8].

### 2.7. Data Extraction

Data were collected in two forms:

1. General data: first author′s name, publication year, sample size (males and females), city, and type of radiography.
2. Main data: DA estimation method, mean and standard deviation of CA and DA, and the difference between DA and CA

To establish standards for data extraction, two reviewers extracted data from the studies separately and entered it into Excel datasheets.

### 2.8. Statistical Analysis

STATA 11 (STATA Corp LP, College Station, Texas, United States) was used to analyze the data. The overall mean of CA and DA, and the MD between CA and DA, with 95% confidence intervals (95% CI), were calculated using a random‐effects inverse‐variance meta‐analysis model. The heterogeneity among the studies was assessed by the chi‐square test and I‐squared (*I* ^2^) statistic. Subgroup analysis by gender (male, female) was also performed. Meta‐regression was used to examine the possible effect of different variables on heterogeneity. The effect of removing individual studies on the overall CA‐DA mean was evaluated by sensitivity analysis. Publication bias was assessed using Begg′s and Egger′s tests, as well as a funnel plot.

## 3. Results

### 3.1. Identification and Selection of Studies

**Figure 1:**
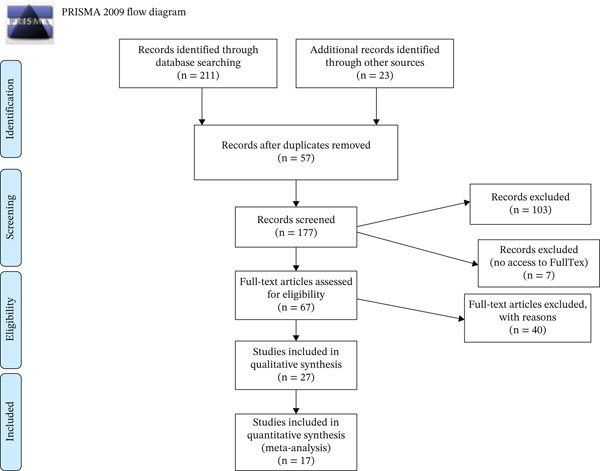
Flowchart of the selection process according to PRISMA in the present meta‐analysis.

We identified 234 potentially eligible records by searching PubMed, Scopus, Web of Science, SID, Magiran, IDM research, and Google Scholar from their inception to 2024. A total of 177 initial records were found after 58 duplicates were removed. Then, the eligibility of the remaining records was evaluated based on their title and abstract, and 103 records were excluded accordingly. A total of 74 studies underwent further eligibility assessment. After full‐text assessment, 47 studies were excluded: 7 due to unavailability of full text, and 40 because they did not report the quantitative parameters required for meta‐analysis, namely the mean and standard deviation of CA and DA (or equivalent data enabling calculation of the mean difference). The process of identifying and selecting studies is presented in Figure 1.

### 3.2. Characteristics of the Studies

The retrieved articles were published between 2009 and 2022 and were conducted in 12 cities/provinces of Iran: Babol (*n* = 4), Isfahan (*n* = 8), Mashhad (*n* = 3), Shiraz (*n* = 3), Kerman (*n* = 3), Qazvin (*n* = 2), Gillan (*n* = 2), Tehran (*n* = 1), and Hamadan (*n* = 1). Demirjian′s method was the most commonly used (17 studies), followed by the Cameriere (three studies), Willem (three studies), Smith (two studies), Nolla (one study), London Atlas (one study), and Moorrees′s method (one study) (Table 2 and Table 3 *).*

**Table 2:** Characteristics of the studies included in the systematic review.

Study	Publication year	Methods	State	Age range	Sample size	Tooth	Main outcome
Namadchian [1]	2022	Cameriere	Babol	5–15	486 panoramic radiographs (208 boys and 278 girls)	Seven left permanent mandibular	Cameriere were not reliable for age estimation in the Iranian population.
Javadi Nejad [36]	1391	Cameriere	Isfahan	5–15	109 panoramic radiographs (42 boys and 67 girls)	Seven left permanent mandibular	Cameriere were reliable for age estimation in the Iranian population specialy female.
Javadinejad [29]	2015	Demirjian, Willem Smith, and Cameriere	Isfahan	3–15	537 panoramic radiographs (264 boys and 273 girls)	Seven left permanent mandibular	Smith′s method to have the highest accuracy among the four assessed methods (Demirjian, Willem Smith, and Cameriere). However, all four methods can be used with acceptable accuracy.
Derayatifar [37]	2018	Demirjian	Mashhad	5–16	235 panoramic radiographs (103 boys and 132 girls)	Seven left permanent mandibular	The dental development age was significantly lower in girls than in boys.
Barati [31]	2022	Demirjian and Willems	Babol	7–15	1320 panoramic radiographs (492 boys and 828 girls)	Seven left permanent mandibular	The Demirjian and Willems methods both overestimated the chronological age. However, the overestimations were smaller in the Willems method.
Kermani [7]	2019	Demirjian	Shiraz	5 to 13	158 panoramic radiographs (77 boys and 81 girls)	Seven left permanent mandibular	Demirjian′s method for determining DA is acceptable but needs to be modified to match the development of teeth in the Iranian population.
Anbiaee [20]	2013	Demirjian	Mashhad	4–15	211 panoramic radiographs (94 boys and 117 girls)	Seven left permanent mandibular	There is a positive relationship between physical status of newborn and the development of permanent teeth.
Javadi Nejad [38]	2014	Demirjian and Willems	Isfahan	3–18	152 panoramic radiographs (47 boys and 105 girls)	Seven left permanent mandibular	The accuracy of Willem′s method is higher than Demirjian′s, and different patterns of tooth development affect the accuracy of these methods and will help the forensic dentist to be more ensured.
Javadi Nejad [39]	2010	Demirjian	Isfahan	7–15	146 panoramic radiographs (57 boys and 89 girls)	Seven left permanent mandibular	Children who were obese had accelerated dental development.
Sheykhi [40]	1390	Demirjian	Babol	5–17	301 panoramic radiographs (118 boys and 183 girls)	Seven left permanent mandibular	Demirjian′s method for determining DA is acceptable in Babol.
Sheykhi [21]	1390	Demirjian	Kerman	5–17	350 panoramic radiographs (224 boys and 126 girls)	Seven left permanent mandibular	Demirjian′s method for determining DA is acceptable in kerman.
Sheykhi [19]	1390	Demirjian	Rasht	5–16	314 panoramic radiographs (102 boys and 212 girls)	Seven left permanent mandibular	Demirjian′s method for determining DA is acceptable in rasht.
Ghafari [2]	2019	London atlas and Smith methods	Isfahan	5–16	339 panoramic radiographs (145 boys and 194 girls)	Seven left permanent mandibular	London atlas and Smith methods had high accuracy for age estimation, but the London Atlas is easier to use.
Kamali‐Sabeti [13]	2018	Demirjian and Nolla methods	Hamedan	5–16	185 panoramic radiographs (76 boys and 109 girls)	Seven left permanent mandibular	The Nolla method is more preferable than the Demirjian method.
Naseh [14]	2016	Moorrees and Demirjian methods	Qazvin	6–15	419 panoramic radiographs (190 boys and 229 girls)	Seven left permanent mandibular	The Moorrees method is not appropriate for Iranian population while the Demirjian method can be acceptable for estimation of dental age based on chronological age.

**Table 3:** Characteristics of the studies included in the meta‐analysis.

Study	Publication year	State	Age range	Sample size	Tooth	CA(MEAN)	CA(SD)	DA(MEAN)	DA(SD)	Male female overall	
1. Abesi [41]	2013	Babol	7–15	168	Seven left permanent mandibular	11.06	2.29	11.44	2.85	Overall	+
				84	Seven left permanent mandibular teeth	11.08	2.31	11.81	2.93	Male	+
				84	Seven left permanent mandibular teeth	11.03	2.28	11.08	2.73	Female	+
2. Bagherian [24]	2011	Kerman	3.5–13.5	519	Seven left permanent mandibular	8.35	2.71	8.54	2.55	Overall	+
				264	Seven left permanent mandibular teeth	8.31	2.68	8.46	2.5	Male	+
				255	Seven left permanent mandibular teeth	8.4	2.73	8.61	2.6	Female	+
3. Bagherpour [25]	2010	Mashhad	6–13	311	Seven left permanent mandibular	9.5	1.75	9.79	1.69	Overall	+
				141	Seven left permanent mandibular	9.53	1.77	9.88	1.65	Male	+
				170	Seven left permanent mandibular	9.47	1.73	9.72	1.73	Female	+
4. Hedayati [42]	2014	Shiraz	6–15	95	Seven left permanent mandibular	11.35	1.94	12.28	2.48	Overall	+
				30	Seven left permanent mandibular	11.59	1.64	12.43	2.6	Male	+
				65	Seven left permanent mandibular	11.24	2.07	12.21	2.44	Female	+
5. Javadinejad [29]	2015	Isfahan	3.9–14.5	537	Seven left permanent mandibular	8.93	2.04	9.8	2.29	Overall	+
				264	Seven left permanent mandibular	8.95	2.07	9.86	2.31	Male	+
				273	Seven left permanent mandibular	8.9	2.01	9.75	2.15	Female	+
6. Naseh [14]	2016	Qazvin	6–15	190	Seven left permanent mandibular	11.24	2.39	11.22	2.53	Male	—
				229	Seven left permanent mandibular	11.28	2.54	11.41	2.64	Female	+
7. Kamali‐Sabeti [13]	2018	Hamedan	5–16	185	Seven left permanent mandibular	9.6	2.8	10	2.8	Overall	+
				76	Seven left permanent mandibular	9.26	3.01	9.68	2.96	Male	+
				109	Seven left permanent mandibular	9.79	2.67	10.21	2.74	Female	+
8. Talaeipour [43]	2022	Tehran	6–14	50	Seven left permanent mandibular	14.02	1.45	14	1.38	Overall	—
				23	Seven left permanent mandibular	14.43	1.25	14.47	1.18	Male	+
				27	Seven left permanent mandibular	13.64	1.25	13.64	1.44	Female	+
9. Movahedian [16]	2018	Shiraz	3–11.99	497	Seven left permanent mandibular	7.82	1.97	8.4	1.94	Overall	+
				222	Seven left permanent mandibular	7.81	1.89	8.6	1.87	Male	+
				275	Seven left permanent mandibular	7.8	2.02	8.22	1.98	Female	+
10. Namadchian [1]	2022	Babol	5–15	486	Seven left permanent mandibular	10.38	2.3	10.67	2.33	Overall	+
				208	Seven left permanent mandibular	10.16	2.24	10.38	2.06	Male	+
				278	Seven left permanent mandibular	10.55	2.33	10.88	2.48	Female	+
11. Eshghi [44]	2011	Isfahan	3–9	221	Seven left permanent mandibular	5.61	1.13	6.2	1.25	Overall	+
				118	Seven left permanent mandibular	5.61	1.15	6.31	1.34	Male	+
				103	Seven left permanent mandibular	5.71	1.11	6.07	1.36	Female	+
12. Javadinejad [45]	1389	Isfahan	6–20	150	Seven left permanent mandibular	10.74	3.02	11.16	2.76	Overall	+
13. Naseh [22]	2016	Qazvin	6–15	468	Seven left permanent mandibular	11.26	2.47	11.32	2.59	Over all	+
14. Javadi Nejad [38]	2013	Isfahan	3–18	152	Seven left permanent mandibular	10.35	3.33	10.86	3.43	Overall	+
15. Tahereh Mohtavipour [15]	2017	Guilan	6–14	184	Seven left permanent mandibular	8.79	1.865	9.61	1.96	Male	+
				206	Seven left permanent mandibular	8.67	1.671	9.51	1.97	Female	+
16. Javadi Nejad [26]	1387	Isfahan	6–14	41	Seven left permanent mandibular	3.9	2	9.3	1.61	Male	+
				63	Seven left permanent mandibular	9.06	1.7	8.9	1.3	Female	—
17. Tafakhori [27]	2016	Kerman	10–15	27	Seven left mandibular permanent teeth	11.93	1.67	11.63	1.9	Male	—
				57	Seven left mandibular permanent teeth	12.79	1.57	12.68	1.84	Female	**_**

### 3.3. Meta‐Analysis

In this meta‐analysis and systematic review, 17 eligible studies that used Demirjian′s method provided sufficient information for meta‐analysis [1, 2, 11–13, 18–20, 23, 28, 31, 36–41]. Inspection of the forest plots revealed that some studies, particularly Javadi Nejad (2008), reported estimates markedly higher than the pooled effect size, thereby contributing to the observed heterogeneity.

#### 3.3.1. Meta‐Analysis of Studies That Used Demirjian′s Method

##### 3.3.1.1. Overall

**Figure 2:**
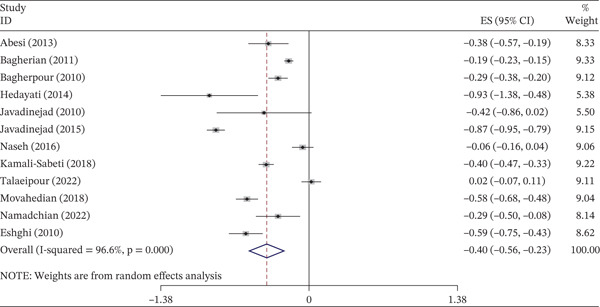
Forest plot of meta‐analysis Demirjian method to determine the difference in the DA versus CA overall.

All included studies were analyzed using pooled effect estimates for males and females aged 3–18 years, and the results are summarized in Figure 2. Considerable heterogeneity among studies was observed in CA (chi − squared = 3648.18, *I* ^2^ = 99.7*%*, tau − squared = 4.68), DA (chi − squared = 2878.48, *I* ^2^ = 99.6*%*, tau − squared = 3.96), and CA‐DA (chi − squared = 324.94, *I* ^2^ = 96.6*%*, tau − squared = 0.072) in the pooled analysis for ages 3–18 years. Overall, the meta‐analysis showed a mean CA of 9.88 years (95% CI: 8.65, 11.11), a mean DA of 10.29 years (95% CI: 9.16, 11.42), and a mean CA–DA of −0.40 years (95% CI: −0.56, −0.23) (Figure 2).

All included studies were analyzed using pooled effect estimates for males and females aged 3–18 years, and the results are summarized in Figure 2.

##### 3.3.1.2. Males

**Figure 3:**
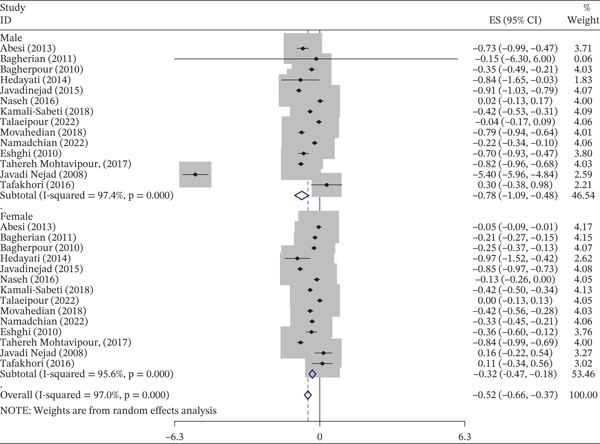
Forest plot of Demirjian′s method for estimation of DA according to the difference between DA and CA based on gender.

The pooled effect estimate for ages 3–20 years in all the included studies is presented in Figure 2. Considerable heterogeneity was found in CA (chi − squared = 3648.18, *I* ^2^ = 99.7*%*), DA (chi − squared = 2878.48, *I* ^2^ = 99.6*%*), and CA‐DA (chi − squared = 324.94, *I* ^2^ = 96.6*%*) in the pooled analysis for ages 3–20 years. Overall, the meta‐analysis showed a significant mean CA of 9.467 years (95% CI: 8.281, 10.653), a mean DA of 10.243 years (95% CI: 9.265, 11.221), and a mean CA‐DA of −0.784 years (95% CI: −1.087, −0.482) (Figure 3).

##### 3.3.1.3. Females

The pooled effect estimates across all included studies were analyzed overall, and the results are summarized in Figure 2. Considerable heterogeneity was found in CA (chi‐squared = 3648.18, *I* ^2^ = 99.7*%*), DA (chi − squared = 2878.48, *I* ^2^ = 99.6*%*), and CA‐DA (chi − squared = 324.94, *I* ^2^ = 96.6*%*) in the pooled analysis for ages 3–20 years. Overall, the meta‐analysis showed a mean CA of 9.875 years (95% CI: 8.800, 10.949), a mean DA of 10.195 years (95% CI: 9.244, 11.147), and a mean CA‐DA of −0.323 years (95% CI: −0.468, −0.178) (Figure 3).

#### 3.3.2. Metaregression

Based on the results of the metaregression, the variables age (*p* = 0.901), study sample size (*p* = 0.828), JBI score (*p* = 0.734), and year of study (*p* = 0.527) did not show a significant relationship with the estimated mean difference in CA‐DA.

#### 3.3.3. Sensitivity Analysis

**Figure 4:**
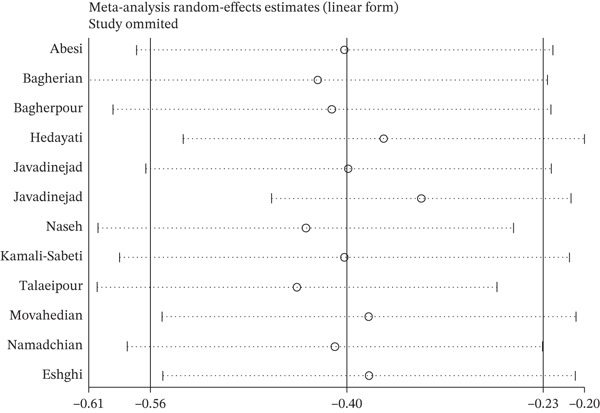
Sensitivity analysis of the effect of removing individual studies on the overall mean estimate of CA‐DA.

The highest CA‐DA mean difference was observed after removing the study by Talaeipour et al. equal to −0.44 years (95% CI: −0.61, −0.27), and the lowest mean difference was observed after excluding the study by Javadinezhad et al. equal to −0.33 years (95% CI: −0.46, −0.21) (Figure 4).

#### 3.3.4. Publication Bias

**Figure 5:**
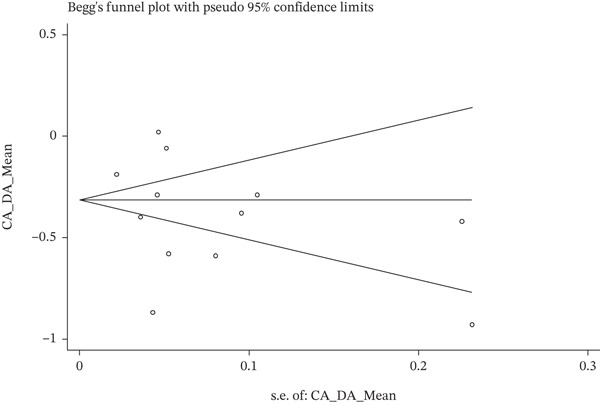
Funnel plot for determining publication bias.

Publication bias was not observed in the primary studies, as indicated by Begg′s (*p* = 0.583) and Egger′s (*p* = 0.382) tests. Moreover, the symmetry between the studies included in the funnel plot indicated the absence of publication bias (Figure 5).

#### 3.3.5. Evaluation of Other DA Estimation Methods

##### 3.3.5.1. Willem′s Method

Only three studies evaluated the accuracy of Willem′s method in Iranian children aged 3–18 years, all conducted in Babol City (2022) [31] and Isfahan (2014 and 2015) [10, 11]. In the three studies above, CA and DA were (10.89 ± 1.98, 10.35 ± 3.33, and 8.93 ± 2.04 years) and (10.97 ± 2.15, 10.14 ± 3.20, and 9.30 ± 2.05 years), respectively, indicating that in studies conducted by Javadinejad et al., in Isfahan (2014 and 2015) [28, 45], Willem’s method underestimated the DA; however, in the study by Torabi et al., in Babol (2022, 31) Willem’s method overestimated the DA. A significant difference was also found in the mean DA and CA across all samples in the two studies [28, 45] (*p* < 0.05), indicating that Willem’s method had low accuracy for age estimation in both males and females.

##### 3.3.5.2. Cameriere′s Method

Only three studies assessed the accuracy of Cameriere′s method for age estimation in children aged 4–15 years; of these, one was conducted in Babol City [1] and two in Isfahan (2012 and 2015) (28, 37). Cameriere′s method overestimated the DA by 0–0.19 years in the studies conducted by Javadinejad et al. in Isfahan [28, 36] and underestimated the DA (by −0.60 years) in the survey by Namadchian et al. in Babol (2022). The underestimation/overestimation was slightly lower in girls (−0.58 to 0.73) than in boys (−0.63 to 0.99). Pearson′s correlation test showed a strong, direct linear relationship between CA and estimated DA.

##### 3.3.5.3. Smith′s Method

Only two studies investigated the accuracy of Smith′s method for age estimation in Iranian children aged 4–16 years, both conducted in Isfahan City (2015, 2019) [2, 10]. They showed that this method overestimated the DA by 0.06 and 0.7 years. The overestimation was slightly higher in girls (0.73–0.73) than in boys (0.12–0.65). A significant difference between CA and DA was not found using this method (*p* > 0.05), indicating that Smith′s method had high accuracy for age estimation in both genders.

##### 3.3.5.4. London Atlas′ method

Only one study investigated the accuracy of the London Atlas′ method for DA estimation in Iranian children between 5 and 16 years, conducted in Isfahan City (2018, 2). DA was overestimated by 0.6 years in this method. The overestimation was slightly higher in girls (0.63 ± 0.58 years) than in boys (0.59 ± 0.56 years). Pearson′s correlation test revealed a strong linear correlation between CA and estimated DA (*p* < 0.001; *r* = 0.96). In addition, a paired *t*‐test showed that the difference between the mean CA and DA was not significant (*p* = 0.15), indicating that the London Atlas method had high accuracy for age estimation.

##### 3.3.5.5. Moorrees′s Method

Only one study investigated the accuracy of Moorrees′s method for DA estimation in Iranian children aged 6–15 years, conducted in Qazvin City (2015) [12]. This method underestimated the DA by −2.45 years. This rate was slightly higher in girls (2.47 ± 1.02 years) than in boys (2.44 ± 0.7 years). Pearson′s correlation test indicated a strong linear correlation between CA and estimated DA (*p* < 0.001; *r* = 0.862). In addition, a paired *t*‐test showed that the difference between the mean CA and DA was significant (*p* < 0.001), indicating that Moorrees′s method was not highly accurate for age estimation.

##### 3.3.5.6. Nolla′s Method

Only one study investigated the accuracy of Nolla′s method for DA estimation in Iranian children aged 5–16 years, conducted in Hamedan City (2018) [14]. This method underestimated the DA by −0.4 years. This underestimation was slightly higher in girls (0.41 years) than in boys (−0.30 years). Pearson′s correlation test suggested a strong linear correlation between CA and DA. In addition, a paired *t*‐test showed that the difference between the mean CA and DA was statistically significant (*p* < 0.001), indicating that Nolla′s method was not highly accurate for age estimation in Iranian children.

## 4. Discussion

This systematic review and meta‐analysis reveals a critical finding: most widely used DA estimation methods demonstrate systematic biases when applied to Iranian children. Although methods like Demirjian′s showed consistent overestimation, others, such as Willem′s, yielded inconsistent results, and a few, such as Smith′s and the London Atlas, showed more acceptable accuracy. This pattern underscores a fundamental principle in forensic and pediatric dentistry: age‐estimation standards are population‐specific and cannot be universally applied without validation. A total of 27 eligible studies were enrolled, which used dental radiography for DA estimation in children [1, 2, 11–15, 18–21, 23–26, 28, 31, 36–45].

The majority of eligible studies (25.92%) were conducted in central Iran, which is the most populous region of Iran; therefore, the reliability of different DA estimation methods was assessed based on dental development in these areas. Most studies (*n* = 17) used Demirjian′s method; given the small number of studies that used other methods, only those that used Demirjian′s method underwent meta‐analysis, and the remaining methods were only systematically reviewed.

When assessed using the GRADE framework, the certainty of the evidence for the overestimation by Demirjian′s method is lowered due to the high heterogeneity and the observational, cross‐sectional nature of the included studies.

### 4.1. Demirjian′s Method

Demirjian′s method is a standard DA‐estimation method based on eight steps of growth and development, from crown and root formation to apex closure, for the seven lower left permanent teeth. Since the difference between CA and DA estimated by this method is insignificant, most studies worldwide and in Iran have used it to estimate DA. In the present study, 17 eligible articles used this method to estimate DA in the Iranian population. The results showed that this method overestimated the DA by 0.4 years [18, 20, 31, 38–40]. In addition, the overestimation rate was greater in boys (0.784) than in girls (0.323). The observed gender differences in DA estimation, particularly the greater overestimation in males than in females when using Demirjian′s method, are biologically plausible and consistent with the existing literature. Females generally exhibit earlier pubertal onset and accelerated dental and skeletal maturation compared with males, largely mediated by hormonal influences, such as estrogen, which plays a role in tooth mineralization and root development. As a result, dental development stages in females may align more closely with CA when using methods derived from mixed‐sex or female‐skewed reference populations.

In contrast, delayed maturation patterns in males may lead to systematic overestimation when applying uniform standards. These findings reinforce the need for sex‐specific calibration of DA estimation methods, particularly in forensic and clinical contexts. Evidence shows that this method overestimates the DA [46, 47], in line with the present findings. A systematic literature review showed that this method significantly overestimated the DA of girls and boys (by 0.62 years) [48], which was in agreement with the present results in the Iranian population. Nonetheless, some studies reported that this method underestimated the DA in the Iranian population. This difference may be due to differences in the age ranges of the populations in the different studies. Moreover, the studies above had relatively small sample sizes, which may explain this difference.

Furthermore, Demirjian′s method was initially developed for a French‐Canadian population, which may explain the differences in results reported by studies conducted in Iran. Since age estimation is fundamental in orthodontic treatment planning and pediatric dentistry, some standards are required to customize this method for each population.

Aside from Demirjian′s method, other DA estimation methods used for the Iranian population were systematically reviewed in this study, among which those by Willem, Cameriere, and Smith were most commonly used.

### 4.2. Willem′s Method

Only three reviewed studies used Willem′s method for DA estimation. One study [31] reported that this method overestimated the DA by 0.07 years, which was consistent with results from studies conducted in other countries, such as Turkey [49], India [50], Malaysia [51], and South Africa [52]. Also, Esan et al. [48] conducted a systematic review and meta‐analysis of Demirjian and Willem′s methods for DA estimation across different populations. They found that Willem′s method overestimated DA in both males and females. Nonetheless, other studies [28, 38] reported that this method underestimated the DA (by 0.2 and 0.36), which was in line with the findings of studies conducted in Turkey [53], Thailand [54], and Sri Lanka [55]. Such variations in different populations can be due to differences in sample size and age range. Moreover, studies [28, 31, 38] showed that this method did not accurately estimate DA in the Iranian population. Nonetheless, Esan et al. [48] reported the high accuracy of this method by reviewing studies worldwide.

Willem′s method demonstrated inconsistent accuracy in the Iranian population, resulting in both under‐ and overestimations. These findings suggest population‐specific calibration may be needed.

### 4.3. Cameriere′s Method

Only three eligible studies used Cameriere′s method for DA estimation, and two reported that it overestimated DA. Pearson′s correlation test revealed a direct, strong correlation between CA and DA estimated by this method. In addition, some studies compared this method with others and demonstrated its high accuracy. Kumaresan et al. [51] compared the validity and reliability of Cameriere′s method with those of the Demirjian, Nolla, Haavikko, and Willems methods for DA estimation in a Malaysian population. They found that Cameriere′s method was more accurate than the others. Moreover, Javadinejad et al. [28] reported, in a study of an Iranian population (3–15 years), that Cameriere′s method had lower accuracy than Smith′s method. Still, its accuracy was higher than that of Willem and Demirjian′s methods. Hostiuc et al. [56] conducted a systematic review and meta‐analysis evaluating Cameriere′s method and reported that it had acceptable accuracy for DA estimation. Nonetheless, this method needs some revisions for accurate DA estimation in each population. There are two main formulae for DA estimation by this method, which are highly similar, and many authors have used them in different populations. Since this method showed acceptable accuracy for DA estimation in most studies, further studies with larger sample sizes and lower heterogeneity are required to reach a final judgment on its reliability and accuracy in an Iranian population.

### 4.4. Smith′s Method

Only two eligible studies used Smith′s method for DA estimation in this review study, and both reported that it underestimated the DA. Smith′s method was highly accurate for DA estimation in both males and females (*p* > 0.05), which was to the results of Corral et al. [57]. Also, considering the high accuracy of this method for DA estimation compared with other methods, such as the Demirjian, Cameriere, and Willems′ methods as reported by Javadinejad et al. [28] and London Atlas′s method as reported by Ghafari et al. [2] as well as its easy application, further studies are required to verify the accuracy and reliability of this method in different populations, particularly in the Iranian population.

### 4.5. Other Methods

Aside from Demirjian, Willem, and Smith′s methods, some other methods, such as the London Atlas, Nolla, and Moorrees′s methods, were also evaluated for use in the Iranian population. However, each technique was assessed only in one study. The results showed that the London Atlas method overestimated DA. In contrast, Nolla and Moorrees′s methods underestimated the DA, and the rate of overestimation/underestimation was greater in females than in males. Nonetheless, only the London Atlas method showed acceptable accuracy for DA estimation among the three methods above. This result, however, is not highly reliable, as only one study was found in this regard. Thus, further studies in different parts of Iran are required to assess further the reliability of these methods for DA estimation, considering the racial differences and their impact on tooth development in children.

This systematic review and meta‐analysis evaluated different methods of DA estimation using radiographs in Iranian children. Many studies were excluded in the second step of the assessment because they did not meet the eligibility criteria, leaving a small number for the systematic review. Nonetheless, the results showed high accuracy of the London Atlas and Smith′s methods for the Iranian population. To further verify the accuracy and reliability of these methods, future studies in different regions of Iran are required to adopt a standard technique based on the dental developmental process of Iranian children for DA estimation. Such methods can be used in forensic dentistry and for treatment planning in orthodontics and pediatric dentistry with higher accuracy.

Although the *I* ^2^ values indicated considerable heterogeneity (above 95%), the *τ* ^2^ statistic also suggested substantial between‐study variability. This heterogeneity could arise from several sources, including regional differences in socioeconomic and nutritional status across Iranian provinces, variations in the age subgroups sampled, differences in radiographic equipment and interpretation, and differences in study design or sample size. These factors should be taken into account when interpreting pooled estimates.

## 5. Limitation

The high statistical heterogeneity observed in our meta‐analysis underscores the significant methodological and demographic variation across studies conducted in Iran, which should be considered when interpreting these pooled estimates.

A limitation of this review is that some included studies sampled orthodontic patients. As this population may not be fully representative of the general pediatric population in terms of dental development, it may introduce selection bias and limit the generalizability of our findings. Additionally, the presence of extreme outliers in certain studies (e.g., Javadi Nejad 2008) may have inflated heterogeneity and influenced pooled estimates. Such deviations may reflect differences in study population characteristics, age distribution, sampling methods, or measurement techniques. Although these studies were retained due to acceptable methodological quality, their findings should be interpreted with caution.

## 6. Future Directions

Future studies should focus on developing customized dental‐age estimation models tailored to specific ethnic and regional populations in Iran. Large‐scale multicenter research can reduce bias and validate the applicability of newer or modified techniques. Additionally, integration of artificial intelligence and machine learning approaches to analyze dental radiographs could enhance precision and reduce interobserver variability.

## 7. Conclusion

Most DA estimation methods studied, including Demirjian′s, tended to overestimate DA, whereas Moorrees′ and Nolla′s methods tended to underestimate age. The inconsistent performance of Willem′s method in the Iranian population, exhibiting both underestimations and overestimations, suggests that population‐specific calibration may be necessary before it can be reliably applied.

The Smith and London Atlas methods, given their promising accuracy, may serve as practical tools in clinical and forensic settings, pending further validation in diverse Iranian populations.

These results emphasize the importance of selecting appropriate DA estimation methods based on their performance in specific populations. Since the Demarjian method is widely used in studies of the Iranian population, customizing it for this group can enhance its relevance. Further research and validation are necessary to encourage professionals to improve and adapt these methods for Iranian children actively. These results highlight the need to select suitable DA estimation methods for specific populations. Customizing the widely used Demarjian method for the Iranian population can enhance its relevance. Further research and validation are crucial to improve these methods for Iranian children.

## 8. Implication for Practice Statement

The findings of this systematic review and meta‐analysis have important implications for dental practitioners, forensic experts, and researchers in the estimation of DA in Iranian children.

## Author Contributions

Conceptualization: A.M., R.J., and M.R.; data curation: A.M. and R.J.; formal analysis: M.R.; methodology: A.M., R.J., and M.R.; writing—original draft: A.M., R.J., and M.R.; writing—review and editing: A.M., R.J., and M.R.

## Funding

No funding was received for this manuscript.

## Disclosure

All the authors have read and approved the final and published version of the manuscript.

## Ethics Statement

The research Ethical committee of the Qazvin University of Medical Sciences approved this study (IR.QUMS.REC.1401.263).

## Conflicts of Interest

The authors declare no conflicts of interest.

## Supporting information


**Supporting Information 1** Additional supporting information can be found online in the Supporting Information section. Graphical abstract

## Data Availability

The data presented in this study are available on request from the corresponding author.
