# Supplementary figures and images for: Accuracy and Reliability of Dental Age Estimation Methods in Iranian Children: A Systematic Review and Meta‐Analysis
Source: Biomed Res Int. 2026 Jun 14;2026:9351167. doi: 10.1155/bmri/9351167 (PMC13265831; doi:10.1155/bmri/9351167)

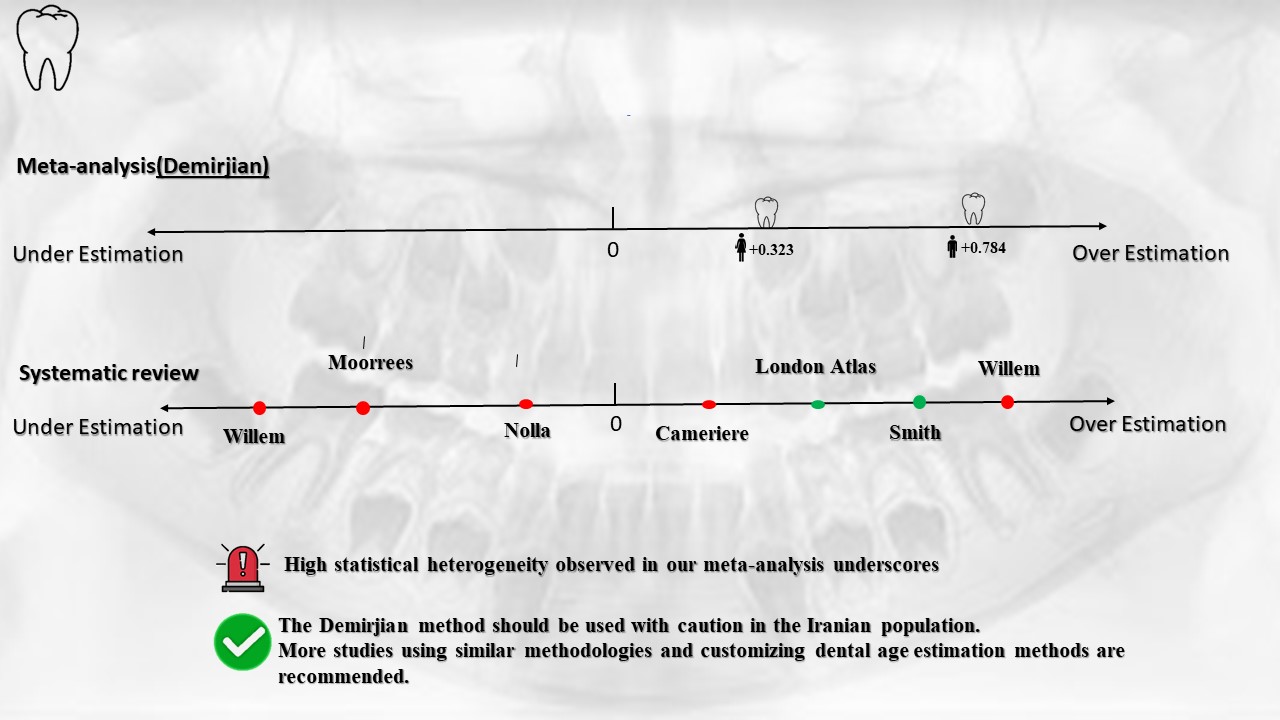

Supplement: Supplementary file 1 — Supporting Information 1 Additional supporting information can be found online in the Supporting Information section. Graphical abstract [file BMRI-2026-9351167-s001.jpg]
